# TIPE2 expression is increased in peripheral blood mononuclear cells from patients with rheumatoid arthritis

**DOI:** 10.18632/oncotarget.21267

**Published:** 2017-09-23

**Authors:** Zhu Shi-Bai, Liu Rui-Min, Sun Ying-Chuan, Zhai Jie, Jiang Chao, Ye Can-Hua, Chen Xi, Qian Wen-Wei

**Affiliations:** ^1^ Department of Orthopedic Surgery, Peking Union Medical College Hospital, Peking Union Medical College, Chinese Academy of Medical Science, Beijing, China; ^2^ Henan University, Kaifeng, China; ^3^ Department of Breast Surgery, Cancer Institute and Hospital, Peking Union Medical College Hospital, Peking Union Medical College, Chinese Academy of Medical Science, Beijing, China

**Keywords:** rheumatoid arthritis (RA), tumor necrosis factor-α–induced protein 8-like 2 (TIPE2), PEST-containing nuclear protein (PCNP)

## Abstract

We investigated the changes in mRNA and protein expression of tumor necrosis factor-α–induced protein 8-like 2 (TIPE2) and PEST-containing nuclear protein (PCNP) in peripheral blood lymphocytes from 54 patients with rheumatoid arthritis (RA) and the spleens of model mice with collagen-induced arthritis (CIA) to generate new ideas for clinical diagnosis and treatment. Expression levels of both TIPE2 and PCNP were higher in RA patients and CIA mice than in their respective controls. They were also higher in the 32 patients with active RA than in the 22 with inactive RA (*P* < 0.001 for both). After comprehensively treating patients with active RA with anti-inflammatory and antirheumatic drugs for 6 months, they were stable, and there was no difference in TIPE2 levels between the treated patients and those with inactive RA (*P* = 0.85). In addition, TIPE2 mRNA levels in peripheral blood correlated positively with PCNP (R^2^ = 0.744, *P* = 0.001). The DAS28 score correlated positively with peripheral blood TIPE2 levels in the RA patients (R^2^ = 0.945, *P* = 0.001). These findings suggest TIPE2 expression increases with the severity of RA.

## INTRODUCTION

Rheumatoid arthritis (RA) is a multisystem inflammatory autoimmune disease that mainly damages peripheral joints, characterized by synovial tissue inflammatory hyperplasia and progressive destruction of articular cartilage [1]. The exact etiology is not clear. The inflammatory process is complex immune and involves a variety of cytokines, adhesion molecules, and chemokines and multigene synergies. However, the exact molecular mechanism of RA remains to be confirmed. Therefore, the identification of new molecules involved in the pathogenesis of RA is essential for development of new treatments.

The tumor necrosis factor-α–induced protein 8-like 2 (TIPE2) gene was discovered in 2002; it helps maintain the self-stabilization of the immune system and is a member of the TIPE family. The encoded protein contains six α-helices and a death effector domain (DED) that can bind to a protein-containing DED domain, such as caspase 8, and plays a role in inhibiting apoptosis [2]. Recent studies have shown that abnormal expression of TIPE2 is associated with inflammatory diseases, particularly autoimmune diseases such as systemic lupus erythematosus (SLE), asthma, and myasthenia gravis [2–5], which suggests TIPE2 may play an important role in the pathogenesis of autoimmune disease by maintaining immune homeostasis.

We hypothesized that TIPE2 contributes to the pathogenesis of RA. To explore its role in RA pathogenesis, therefore, we assess TIPE2 mRNA expression and the relationship between TIPE2 expression and RA disease activity score (DAS28).

PEST-containing nuclear protein (PCNP) is a protein found mainly in the nucleus. A new type of finger protein, PCNP is a protein ligase with ubiquitination ability, and is involved in protein degradation via the ubiquitination pathway [6]. Studies have shown that PCNP is associated with cell-cycle regulation. The relationship between regulator molecules, such as PCNP and TIPE2, and RA is rarely investigated, so we also analyzed the relationship between TIPE2 and PCNP.

## MATERIALS AND METHODS

### Subjects

Fifty-four RA patients (38 women and 16 men; average age, 46 ± 10 years) who were treated in the Department of Rheumatism at the First Affiliated Hospital of Henan University were enrolled. Disease course ranged from 4 to 10 years. RA patients were divided into two groups according to DAS28 score: active group (DAS28 > 3.2, *n* = 32) and inactive group (DAS28 ≤ 2.6, *n* = 22). A control group of 30 healthy subjects was chosen during the same period and matched for age and sex (24 women and 16 men; average age, 44 ± 9 years). Healthy controls had no history of autoimmune disease or genetic susceptibility. All subjects provided informed consent.

### RA DBA1/j mice models

DBA1/j mice (male, 6 weeks old, purchased from Beijing HFK Bioscience Co., Ltd., Institute of Laboratory Animal Sciences, Chinese Academy of Medical Sciences) were randomly divided into a model group and control group. Bovine type II collagen (Chondrex, Redmond, WA) was dissolved in 2 mL of 0.05 M glacial acetic acid solution at a final concentration of 2 mg/mL. This solution was mixed with an equivalent volume of Freund's complete adjuvant, and the final concentration of collagen was 1 mg/mL. Approximately 100 μL of mixed emulsion was injected into the tails of mice in the model group on day 1 and then again 3 weeks later. The same method was adopted to inject 100 μL of emulsion containing Freund's complete adjuvant and equivalent volume of 0.05 M glacial acetic acid into the mice in the control group. Collagen-induced arthritis (CIA) clinical manifestations were observed, and an inflammatory index was scored in mice every other day after day 1, as follows: no foot swelling = 0 points; slight redness of toe = 1 point; moderate swelling of foot and toe joint = 2 points; whole-foot swelling accompanied by inflammation = 3 points; and whole-foot swelling and inflammation and deformation of toe joint = 4 points. Each limb score was added to the total score of joint inflammation.

### Detection of TIPE2 and PCNP genetic expression in mice

TRIzol method (Invitrogen, Carlsbad, CA) was used to extract total RNA from splenic lymphocytes in CIA mice. The obtained splenic cDNA was diluted to 100 ng/μL, with β-actin as the internal reference; primer sequences were 5′-TTGTCAGCAATGCATCCTGCAC-3′ (upstream) and 5′-ACAGCTTTCCAGAGGGGCCATC-3′ (downstream), and the amplified product was 399 bp. Amplification of TIPE2 gene fragment primers were 5′-AATACACGCACAGCCGGCCC-3′ (upstream) and 5′-CGGCACTCGACGAGCAGACC-3′ (downstream), and the amplified product was 229 bp. Amplification of PCNP gene fragment primers were 5′-CAGAGAAG CCGCAGCGAGCC-3′ (upstream) and 5′-GGACGCTTTC CGTGCCGTCT-3′ (downstream), and the amplified product was 221 bp.

### Real-time quantitative polymerase chain reaction

Density gradient centrifugation was used to isolate peripheral blood mononuclear cells (PBMCs) from peripheral blood anticoagulated by sodium citrate. Cellular sediment was cleaved with TRIzol reagent (Invitrogen). Total RNA was extracted according to the manufacturer's instructions, and the obtained RNA was transcribed into cDNA. Fluorescence real-time quantitative polymerase chain reaction (PCR) was used to detect expression of the TIPE2 gene in freshly isolated lymphocytes of patients and healthy controls. The amplified product of internal reference, β-actin, was 220 bp, the target fragment of TIPE2 was 214 bp, and the primer sequences were 5′-GCCCAGCGCGTATCAAGGA-3′ (upstream) and 5′-GCACATCCCGGCACTCGGTC-3′ (downstream). The reaction conditions were as follows: pre-denaturation 95°C for 10 minutes; 95°C for 30 seconds, 56°C for 30 seconds, 72°C for 30 seconds, 35 circular reactions; and 72°C to 95°C melting curve analysis. PCNP gene expression in freshly isolated lymphocytes was also detected using fluorescence real-time quantitative PCR, and the amplification primers of β-actin were 5′-CCTAGAAGCATTTGCGGTGG-3′ (upstream) and 5′-GAGCTACGAGCTGCCTGACG-3′ (downstream), and the target primer was 220 bp. The amplification primers of PCNP were 5′-GACGCTTTCCGTGCCGTCTGAC-3′ (upstream) and 5′-AGCCGCCGGAGGACCTGAAGA-3′ (downstream), and the target primer was 224 bp. The reaction conditions were as follows: pre-denaturation 95°C for 10 minutes; 95°C for 30 seconds, 56°C for 30 seconds, 72°C for 30 seconds, 35 circular reactions; and 72°C to 95°C melting curve analysis. Experimental results were analyzed by Rotor-Gene 6000 Series Software in the fluorescence quantitative PCR instrument.

### Western blot for detecting differences in expression of TIPE2 protein

Lymphocytes were collected from spleens of CIA mice and peripheral blood of patients and healthy controls. Proteins were extracted with lysate. SDS-PAGE electrophoresis was performed on protein extraction samples. The concentration of spacer gel was 5%, and the concentration of separation gel was 10%. The samples were transferred to PVDF membrane after electrophoresis, adding rabbit anti-human anti-mice TIPE2 antibody and mice anti-human β-actin antibody, and staying overnight at 4°C. Then, samples were washed three times with PBST, incubated for 1 hour with sheep anti-rabbit secondary antibody at room temperature, washed four times with PBST, and treated with membrane washing three times. Samples were then subjected to color development for 1 minute in ECL reaction solution and tableting imaging in a dark room. β-Actin was considered as the internal reference to detect consistency of loading amount. The same method was used to detect expression of PCNP protein.

### Statistical analysis

The data collected by fluorescence quantitative PCR was statistically analyzed using SPSS 19.0 software. The measurement data were expressed as mean ± standard deviation (x ± s). The total difference was compared using one-way analysis of variance (ANOVA), comparison between the two groups was done using the least significant difference test (LSD-t), and correlation analysis was performed using the Spearman rank correlation method. *P* < 0.05 was considered statistically significant.

## RESULTS

### CIA mice model

**Figure 1:**
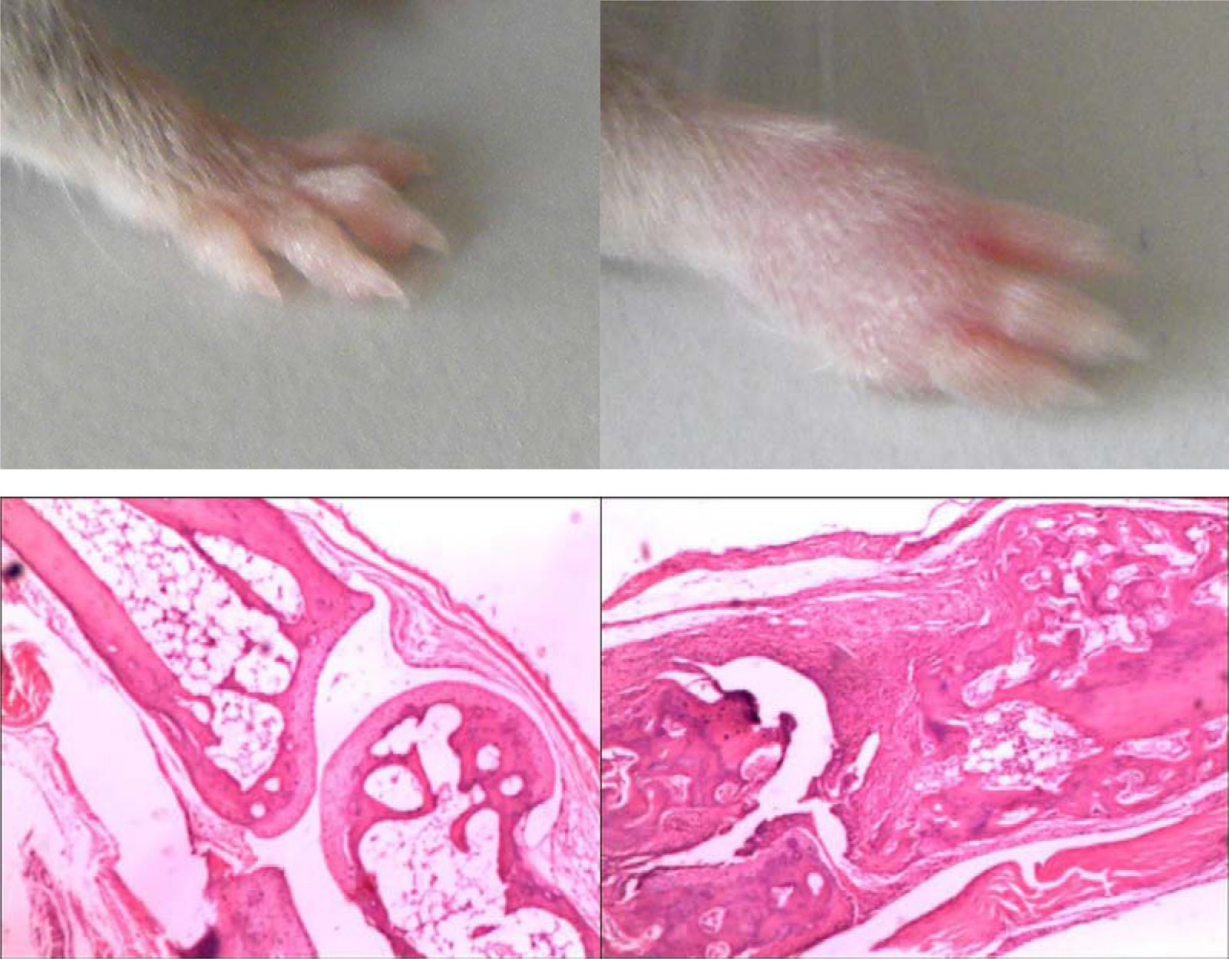
Histologic analysis in the joints of hind limbs in mice with CIA (**A**) Representative joint sections from PBS-treated normal mice stained with hematoxylin and eosin (H&E). (**B**) Representative joint sections from arthritic mice stained with H&E.

After 28 days of immunization with bovine type II collagen, the toes of DBA1/j mice showed obvious swelling of pharyngeal joints, metacarpal joints, and proximal and distal interphalangeal joints; the swelling reached a peak at about 40 days. Hematoxylin and eosin staining of joints showed significant inflammatory cell infiltration, synovial hyperplasia, and bone destruction in the CIA mice model (Figure 1). CIA was successfully induced in the DBA1/j mice model. Post-claw joints were normal in phosphate-buffered saline (PBS) control mice.

### PCR results

### Reverse transcriptase PCR results of TIPE2 and PCNP in mice

**Figure 2:**
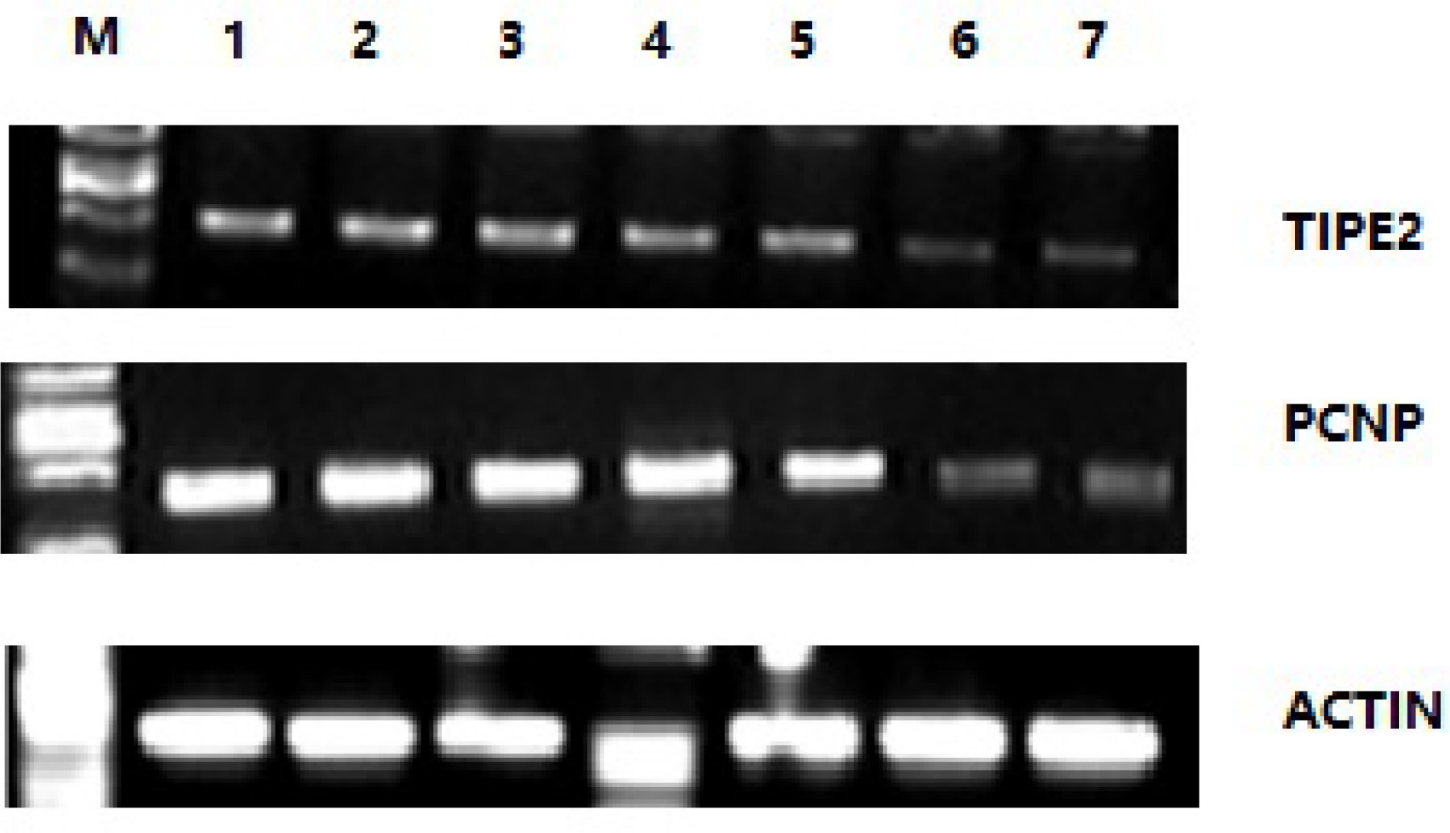
Reverse transcriptase PCR results of TIPE2 and PCNP in spleen cells of CIA mice and normal mice M is the marker DL2000; 1 to 5 present the quantity of expressed genes in five CIA mice; and 6 and 7 are the quantity of expressed genes in the control groups. Gene expression in CIA mice groups is significantly greater than that in the control groups.

Semiquantitative PCR was used to detect TIPE2 and PCNP expression, using cDNA in spleens of CIA mice and normal mice as reference. The electrophoretic results are shown in Figure 2. The results showed that the banding brightness of TIPE2 and PCNP in CIA mice was brighter than that in normal mice, which indicated that TIPE2 and PCNP gene expression in spleens of CIA mice was higher than that in the normal control group.

### Quantitative PCR analysis of TIPE2 and PCNP in peripheral blood of RA patients

**Figure 3:**
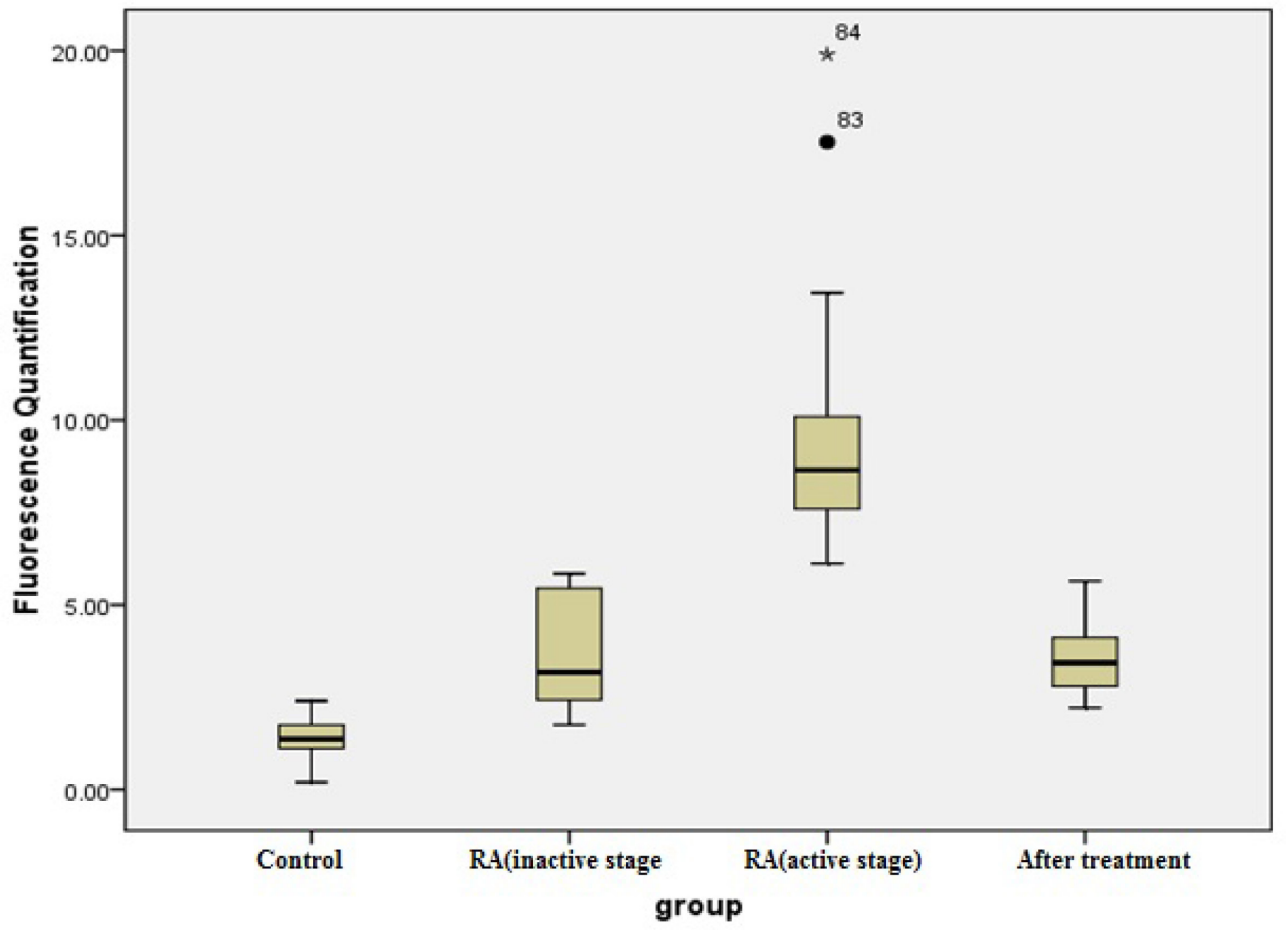
TIPE2 relative expression levels Activate RA versus inactive RA, ^*^P < 0.001; active RA versus treated RA, ^#^*P* < 0.001; inactive RA versus treated RA, *P* = 0.85.

**Figure 4:**
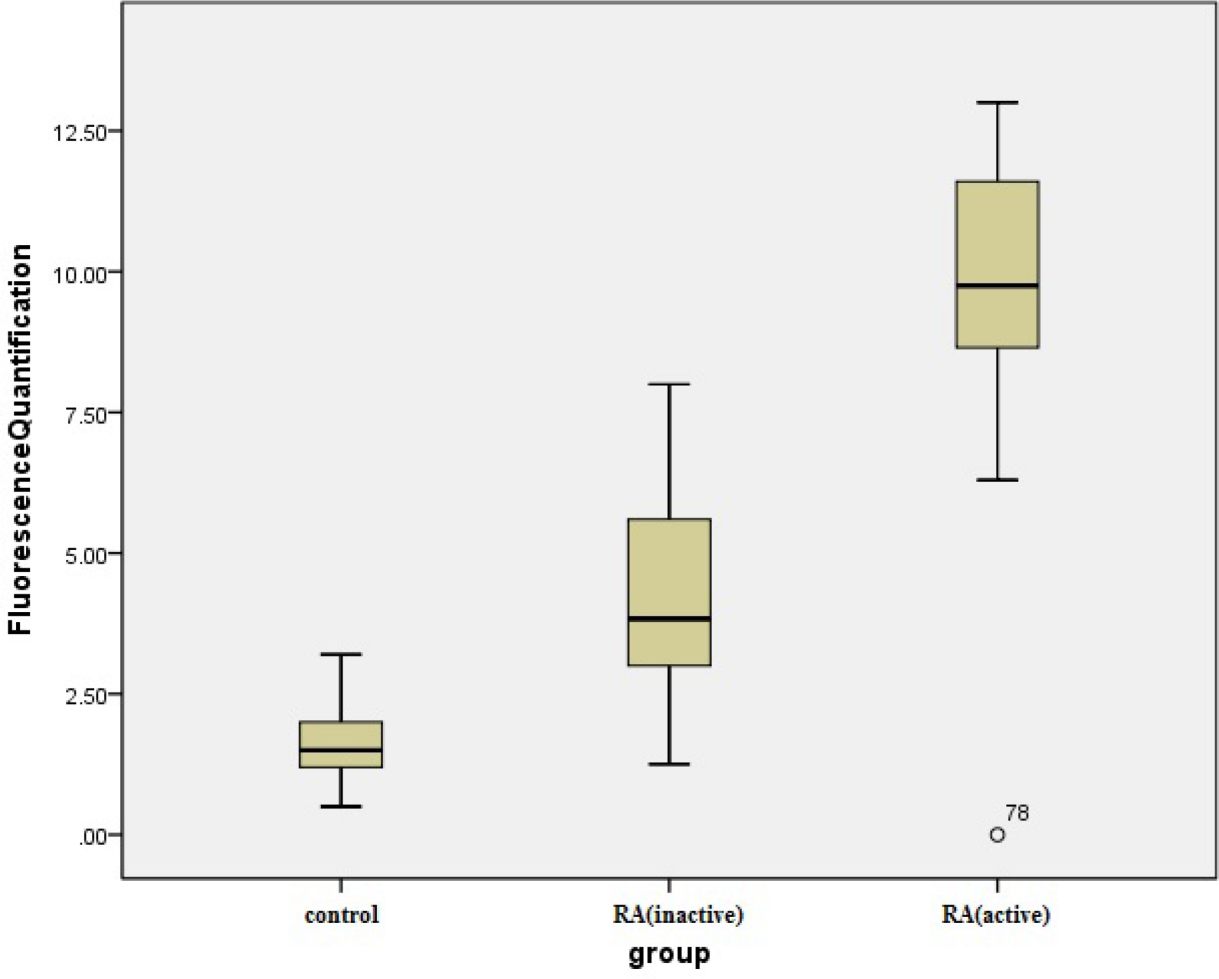
Comparison of the PCNP relative expression levels in patients with inactive and active RA Active RA and inactive RA versus control group, *P* < 0.001; active RA versus inactive RA, ^*^P < 0.001.

Quantitative PCR showed that expression levels of TIPE2 were 2^−ΔΔCt^ (3.62 ± 1.42) and 2^−ΔΔCt^ (9.42 ± 3.01) in peripheral blood of patients with inactive RA (*n* = 22) and active RA (*n* = 32), respectively, both of which were higher than the level observed in the healthy control group (1.43 ± 0.49; *n* = 30). Thirty-two patients with active RA became stable after comprehensive treatment with analgesic anti-inflammatory and antirheumatic drugs for 6 months, reducing the expression level of TIPE2 in peripheral blood to 2^−ΔΔCt^ (3.53 ± 0.89; ANOVA, F = 116.12, *P* < 0.001). LSD-t showed a significant difference among the RA active, RA inactive and RA treated groups (*P* < 0.001) but not between the RA inactive and RA treated groups (*P* = 0.85; Figure 3). PCNP expression levels in peripheral blood of patients with inactive and active RA were 2^−ΔΔCt^ (4.13 ± 1.59) and 2^−ΔΔCt^ (9.64 ± 2.55), respectively, and significantly higher than that in the healthy control group (1.55 ± 0.58; one-way ANOVA, F = 161.65.12, *P* < 0.001). LSD-t showed a statistically significant difference between the RA active and RA inactive groups (*P* < 0.0001; Figure 4).

### Western blot results

**Figure 5:**
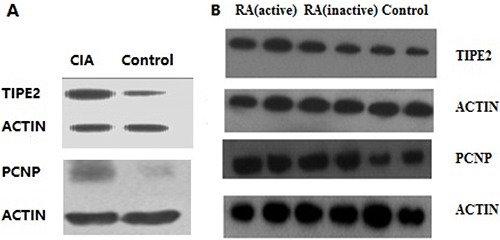
Western blot results (**A**) The left column shows binds of proteins expressed in CIA mice; the right column shows binds of proteins expressed in the control group. Protein expression in CIA mice was significantly greater than that in the control group. (**B**) There are three groups of human lymphocytes. Protein expression in the RA groups was significantly greater than that in the control group.

The expression levels of TIPE2 and PCNP proteins in the experimental group were significantly higher than those in the control group by Western blot. The results are shown in Figure 5A. The expression levels of TIPE2 and PCNP proteins in peripheral blood of RA patients were higher in those with active disease than those with inactive disease, and the latter were higher than the levels in the normal control group.

### Positive correlation between DAS28 score and TIPE2 expression in RA patients

**Figure 6:**
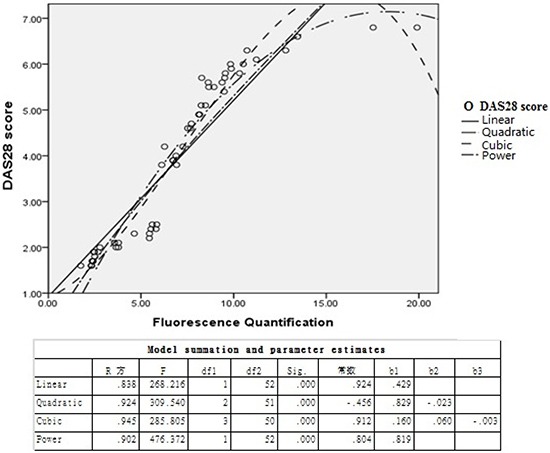
Correlation analysis between TIPE2 expression level and DAS28 score in RA patients

Clinical manifestations and laboratory abnormalities of 54 RA patients were analyzed according to DAS28 scores, and the results showed a significant positive correlation between DAS28 score and TIPE2 expression in peripheral blood of RA patients (R^2^ = 0.945, *P* = 0.001), which was identical to the cubic model (Figure 6).

### Correlation between TIPE2 and PCNP expression

**Figure 7:**
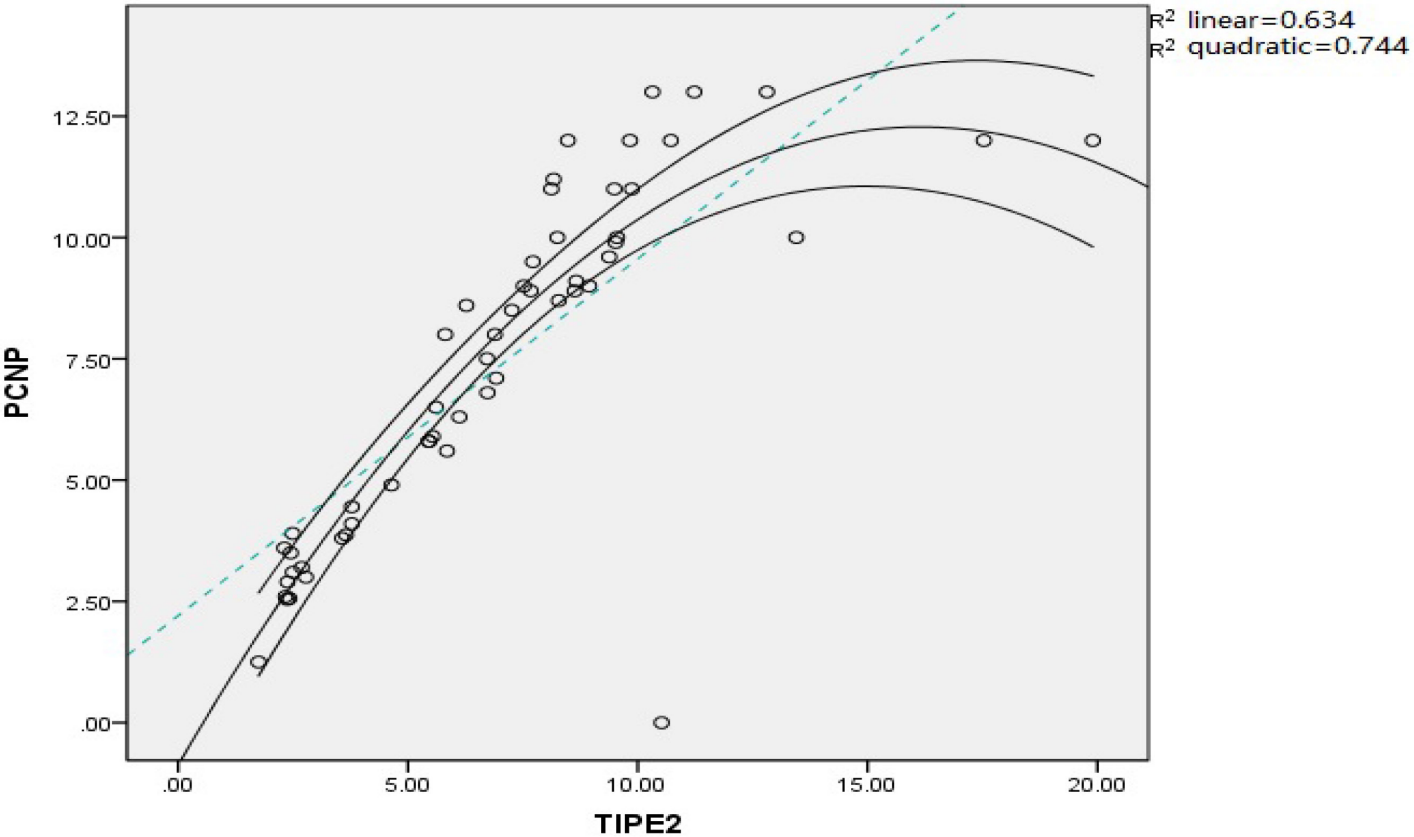
Correlation analysis between TIPE2 and PCNP expression in RA patients

Correlation between TIPE2 and PCNP expression in peripheral blood was analyzed. The results showed a positive correlation between TIPE2 and PCNP expression (R^2^ = 0.744, *P* = 0.001), which was identical to the quadratic model (Figure 7).

## DISCUSSION

RA is a chronic progressive autoimmune disease with pathologic features that include synovial hyperplasia and inflammatory cell infiltration of the joints. Clinical manifestations include joint swelling, pain, rigidity, and deformity and severely limited joint function in late-stage disease, and the prognosis is often poor [1, 3]. Treatment of RA is currently bsuboptimal, so it is important to clarify the specific pathogenic mechanisms of RA. A large number of immune function-related regulatory genes play important roles in the pathogenetic process of RA, and the content of some regulatory genes changes significantly in immune cells.

TIPE2 is a negative regulatory molecule involved in maintaining immune homeostasis [2]. It was first discovered in 2002 by Chen and colleagues at the Pennsylvania State University in experimental autoimmune encephalomyelitis mouse models [2–3]. In the present study, our results showed that TIPE2 expression is increased in patients with RA and that TIPE2 expression is positively correlated with DAS28 score in RA patients. As a negative regulator of T cells and macrophages, TIPE2 can inhibit inflammation in autoimmune diseases. Previous studies showed that TIPE2 mRNA expression was lower in PBMCs of SLE patients than normal controls and that TIPE2 expression correlated negatively with SLE disease activity [7]. On the other hand, TIPE2 mRNA expression was increased in patients with acute chronic hepatitis B liver failure or ulcerative colitis [8]. These results suggest that abnormal expression of TIPE2 may play an important role in the pathogenesis of inflammatory disease, particularly in autoimmune diseases. In this study, increased expression of TIPE2 in PBMCs from RA patients may be caused by activated T cells and macrophages. Our results also showed that after successful RA treatment, TIPE2 expression was reduced. We also successfully established an RA mouse model through bovine type II collagen immunization of DBA/j mice. Using this model, we found that TIPE2 expression in spleen cells of RA mice was higher than in control mice. Animal experiments also confirmed a close relationship between TIPE2 and onset of RA. However, specific mechanisms of TIPE2 involvement in pathogenesis of RA need to be confirmed in further studies.

Previous studies showed that TIPE2 can inhibit inflammation and plays an important role in inflammation and cell repair [9]. It has also been shown that lipopolysaccharide can stimulate secretion of inflammatory cytokines such as tumor necrosis factor-α and interleukin-6 in RAW264.7 cells, but this process could be inhibited by TIPE2 [10]. In addition, secretion of inflammatory cytokines increased in TIPE2-deficient mice treated with lipopolysaccharide [2]. These data suggest that TIPE2 inhibits the immune response at the time of inflammation. Interestingly, we found that increased TIPE2 expression correlated positively with increased PCNP expression. PCNP is a protein found predominantly in the nucleus. A novel finger protein, PCNP is a ubiquitination-capable protein ligase involved in ubiquitination pathway of protein degradation. Studies show that it correlates with cell-cycle regulation [6, 7]. The role of TIPE2-mediated increases in PCNP in RA is presently unclear.

In conclusion, RA increases the expression of TIPE2, which positively correlates with DAS28 score in RA patients, suggesting that TIPE2 measurement can be used as a simple method to evaluate RA activity in clinical trials. However, further research is needed in view of the limited available data. More research is needed on how TIPE2 participates in the pathology of RA and the basic mechanism of TIPE2 in RA.
